# Teaching Medical Students to Help Patients Manage Their Weight: Outcomes of an Eight-School Randomized Controlled Trial

**DOI:** 10.1007/s11606-020-06571-x

**Published:** 2021-04-09

**Authors:** Judith K. Ockene, Lori Pbert, Sybil Crawford, Christine F. Frisard, Jyothi A. Pendharkar, Rajani S. Sadasivam, Jamie Faro, Cathy Okuliar, Cassie Eno, Katherine Margo, Monica Ann Shaw, Taraneh Soleymani, Diane D. Stadler, Sarita Warrier, Katie White, Alan C. Geller

**Affiliations:** 1 Department of Population and Quantitative Health Sciences, Division of Preventive and Behavioral Medicine, University of Massachusetts Medical School, 368 Plantation Street, Worcester, MA 01605 USA; 2 Georgetown University Hospital, Washington, DC USA; 3 Creighton University School of Medicine, Omaha, NE USA; 4 Perelman School of Medicine, University of Pennsylvania, Philadelphia, PA USA; 5 University of Louisville School of Medicine, Louisville, KY USA; 6 Previously at the University of Alabama, Birmingham, AL and now at Penn State Health, Middletown, PA USA; 7 Oregon Health and Science University, Portland, OR USA; 8 Brown University, Providence, RI USA; 9 University of Iowa, Iowa City, IA USA; 10 Department of Social and Behavioral Sciences, Harvard School of Public Health, Cambridge, MA USA

**Keywords:** weight management counseling, medical school curriculum, medical student behaviors, randomized controlled trial, objective structured clinical examination

## Abstract

**BACKGROUND:**

Given the rising rates of obesity there is a pressing need for medical schools to better prepare students for intervening with patients who have overweight or obesity and for prevention efforts.

**OBJECTIVE:**

To assess the effect of a multi-modal weight management curriculum on counseling skills for health behavior change.

**DESIGN:**

A pair-matched, group-randomized controlled trial (2015-2020) included students enrolled in eight U.S. medical schools randomized to receive either multi-modal weight management education (MME) or traditional weight management education (TE).

**SETTING/PARTICIPANTS:**

Students from the class of 2020 (N=1305) were asked to participate in an objective structured clinical examination (OSCE) focused on weight management counseling and complete pre and post surveys. A total of 70.1% of eligible students (N=915) completed the OSCE and 69.3% (N=904) completed both surveys. INTERVENTIONS: The MME implemented over three years included a web-based course, a role-play classroom exercise, a web-patient encounter with feedback, and an enhanced clerkship experience with preceptors trained in weight management counseling (WMC). Counseling focused on the 5As (Ask, Advise, Assess, Assist, Arrange) and patient-centeredness.

**MEASUREMENTS:**

The outcome was student 5As WMC skills assessed using an objective measure, an OSCE, scored using a behavior checklist, and a subjective measure, student self-reported skills for performing the 5As.

**RESULTS:**

Among MME students who completed two of three WMC components compared to those who completed none, exposure was significantly associated with higher OSCE scores and self-reported 5A skills.

**LIMITATIONS:**

Variability in medical schools requiring participation in the WMC curriculum.

**CONCLUSIONS:**

This trial revealed that medical students struggle with delivering weight management counseling to their patients who have overweight or obesity. Medical schools, though restrained in adding curricula, should incorporate should incorporate multiple WMC curricula components early in medical student education to provide knowledge and build confidence for supporting patients in developing individualized plans for weight management.

**NIH Trial Registry Number:**

R01-194787

## INTRODUCTION

Obesity (BMI ≥ 30 kg/m2) is a serious chronic disease and a major contributor to the global burden of cancers, heart disease, type 2 diabetes, liver disease, sleep apnea and now COVID-19.^1–3^. In the U.S., the prevalence of obesity has reached epidemic proportions; 42.4% of adults have obesity^4^. Although physicians are essential in helping patients manage their weight, many struggle with providing effective counseling to patients with weight challenges ^5, 6^.

According to an analysis of 5,054 participant responses in the National Health and Nutritional Examination survey (NHANES), patients whose physicians discussed weight loss with them reported clinically significant weight loss^7^. However, only 20–40% of adult patients with obesity receive weight management counseling (WMC) from a physician^8–11^. Given evidence that physicians can help patients manage their weight,^12–18^ the U.S. Preventive Services Task Force (USPSTF) gives physician-delivered weight management interventions (e.g., 5A counseling: Ask, Advise, Assess, Assist, Arrange) a “B” recommendation, indicating physicians should “screen all adult patients” and “offer intensive counseling and behavioral interventions.”^19^

Given the importance of weight loss counseling with patients, medical schools have incorporated it into their curricula; however comprehensive medical school curricula combining didactics, interactive counseling practice using role-play or standardized patients, and observed clinical experiences with patients who have weight challenges are not widespread. Weight management is often part of discussions of other diseases, with no standard stand-alone curriculum for WMC. Medical students need practice to develop WMC skills, yet a recent survey of US medical schools reports that many schools fail to prepare future physicians for everyday nutrition challenges in clinical practice^20^.The Association of American Medical Colleges(AAMC) recommends medical schools address this deficit by emphasizing WMC in their curricula.^21^

The goal of “MSWeight” (Medical Students learning WMC skills) was to develop, implement, and evaluate a comprehensive multi-modal education (MME) intervention for teaching medical students effective WMC. We hypothesized that MME students receiving the intervention in years 1-3 would demonstrate a marked improvement in their WMC skills as compared to students receiving traditional education (TE) which represents “usual care”.

## METHODS

### Trial Design

Eight U.S. medical schools were pair-matched and randomized to an MME or TE curriculum starting in the first year (MS1). The outcome was demonstration of WMC skills in the third year (MS3) assessed through a weight management-specific Objective Structured Clinical Examination (OSCE), and self-reported skills in performing the 5As. We compared scores for MS3 students in MME and TE schools.

### Participating Schools

Schools met the following criteria: 1) at least 90 MS1s; 2) ≤4 hours of WMC education during the first 3 years of medical school curriculum; 3) offer a family medicine or internal medicine clerkship for MS3s; and 4) include an evaluation of MS3s using a standard OSCE and willing to include the study WMC case. Institutional review boards at each participating medical school approved the study. The U.S. schools were private (N=4) or public (N=4) in geographically diverse regions.

### Pair-Matching and Randomization

Before randomization, principal investigators at each of the eight schools were surveyed about the number of hours of curricula addressing behavior change and WMC during MS1–MS3 in their medical schools^22^. Questions on this survey were based on content and format from previous research. In addition, a Matching and Randomization survey was administered to the Class of 2017 in both TE and MME schools which assessed various variables, including student self-perceived WMC skills (16 items; responses 1=not at all skilled, to 5=very skilled )^22^. Schools were stratified into high (four schools) or low (four schools) and these pair-matched schools were randomly assigned to MME or TE.

### Educational Interventions

#### MME: Multi-Modal Education

The MME intervention included components implemented during the MS1–MS3 years: 1) a 4-hour self-paced web course that is evidence-supported, competency-based and covers all 5As, emphasizing Assist and Arrange (MS1); 2) a role-play exercise in tandem with the Web course (MS1); 3) a web-patient encounter^22^ with feedback (MS2); and 4) an enhanced clerkship working with trained preceptors (MS3) and patients with overweight/obesity as part of their regular clerkship. Preceptors from family and internal medicine clerkships attended a 30–60-minute individual or group session reviewing WMC guidelines, and how to model, observe, instruct, and provide critical feedback to students regarding the 5As. Each MME component included written instructions, objectives, and discussion points to ensure students received the intervention in a standardized manner across MME schools.

#### MME Theoretical Framework

MSWeight is a multi-modal educational intervention (MME) guided by Social Cognitive Theory,^23^, and Socio-Ecological Theory ^24^. The curriculum was designed to address key theoretical constructs at the individual, inter-personal, and institutional level hypothesized to influence WMC skills ^22^.

The Web course is intended to help students develop positive attitudes toward WMC. The role-play exercise and standardized WebPatientEncounter^22^ provides practice and structured feedback on the students’ WMC skills, and the preceptor facilitated teaching during an enhanced clerkship provides opportunities for observation, instruction and feedback. We hypothesized that together these curriculum elements would provide a structured foundation to help medical students build and practice WMC skills as well as reinforce confidence in their skills and ability to implement WMC.

#### TE: Traditional Education

The four TE schools were instructed to continue delivering their existing curriculum regarding WMC during years 1 through 3. Based on a survey of the schools’ investigators, this consisted of less than 4 hours of WMC-related content (e.g., obesity, nutrition, behavior change, communication skills) and included largely didactic teaching interspersed among basic science and behavioral counseling classes during years 1 and 2, with some small group discussions or skill-building exercises and clinical experiences for health beahaviors^22^^,^

### Outcome Measures

We assessed WMC skills using an OSCE, a standard objective method to evaluate medical students in the U.S. The WMC OSCE case and associated checklist were developed by the investigators specifically for this study based on the 5As counseling approach recommended by the USPSTF.^22, 25^ OSCEs were implemented within the context of existing clinical skills assessments, videotaped, then scored by a trained rater blinded to the student’s school. The 15-item behavior checklist assessed multiple aspects of each 5A behavior, scored Yes/No (range of 0-15) (Table 1). A ‘gold-standard’ rater who was extensively trained by the checklist developers reviewed and coded a random sample of 10% of all completed checklists. The discrepancy rate between coders and the gold-standard rater on this 10% random sample was 3.4% which was below our pre-determined 5% threshold.

**Table 1.** 5As OSCE Scores and Individual OSCE Items by Randomization Assignment

**SCORES**	**Mean (SE)** **MME (N=479)**	**Mean (SE)** **TE (N=436)**	**P-Value**
Total number of items performed: (Range 0 – 15)	8.7 (0.11)	8.2 (0.11)	0.16
	**% of Students Performing Each Item**		
	**MME** **N=479**	**TE** **N=436**	**P-Value**
**ASK**			
Reviewed medical risk factors with the patient	91.2%	88.7%	0.44
Discussed weight history and prior weight loss experience	24.6%	19.7%	0.30
Asked about current diet and dietary habits.	85.2%	90.1%	0.37
Discussed current level of physical activity.	88.7%	90.7%	0.50
**ADVISE**			
Shared BMI/concerns related to weight with patient	34.4%	33.4%	0.91
Advised that weight loss is recommended	26.4%	25.5%	0.86
Provided information on health benefits of losing 3-5% of current weight	11.1%	4.6%	0.09
**ASSESS**			
Assessed patient's level of motivation/commitment/readiness to make changes	69.4%	66.5%	0.71
Assessed patient's level of confidence/self-efficacy	69.4%	59.1%	0.22
**ASSIST**			
Discussed perceived barriers and concerns	97.3%	95.8%	0.30
Provided relevant information regarding relationship between weight, diet, and physical activity.	9.8%	7.3%	0.40
Partnered with the patient to encourage development of specific goals and plans	78.4%	70.1%	0.16
Assisted the patient by discussing behavior change strategies	99.1%	98.6%	0.66
**ARRANGE**			
Recommended or referred the patient to weight management resources	41.4%	48.3%	0.50
Proposed that weight and weight management be discussed again at the patient's next appointment.	58.9%	47.9%	0.24

We also assessed WMC skills using a subjective measure of student self-reported perceived skill level for each of the 16 5A items assessed in the OSCE, using a 4-category Likert scale (Table 2). Students completed a baseline survey as MS1s and a follow-up survey as MS3s. Total score was calculated as the average of the16 items.

**Table 2.** Student Self-Report of Weight Management Counseling Skills by Randomization Assignment

	**MME**	**TE**	
	**Mean (SE)** **N=474**	**Mean (SE)** **N=432**	**p-value**
Total Perceived WMC skills score (Range 1 – 4)	3.18 (0.06)	3.09 (0.06)	.25
	**% Moderately/ very skilled (N)**	**% Moderately/ very skilled (N)**	**P-Value**
Sharing with the patient their BMI and BMI classification	77.3% (344)	70.4% (289)	0.45
Identifying the patient's medical risk factors and co-morbidities of obesity	90.5% (404)	89.5% (366)	0.66
Assessing the patient's prior weight loss experiences	81.8% (347)	77.7% (301)	0.44
Assessing the patient's current diet and dietary habits	86.4% (386)	86.8% (355)	0.89
Assessing the patient's current level of physical activity	89.2% (405)	87.9% (368)	0.67
Advising weight loss based on their personal health information (e.g. BMI and risk factors)	80.5% (342)	74.2% (282)	0.02
Discussing with the patient the health benefits of losing about 5% of their current weight	73.8% (287)	61.0% (212)	0.06
Assessing the patient’s level of readiness to make lifestyle changes to achieve weight loss	83.6% (359)	81.0% (315)	0.51
Identifying and discussing with the patient their perceived barriers and concerns that make it hard to lose weight	84.8% (368)	82.4% (335)	0.62
Partnering with the patient to encourage development of their own set of goals and specific plans based on their interests and willingness to change behavior	84.1% (363)	84.2% (334)	0.99
Assisting the patient by providing information regarding the relationship between weight, diet and physical activity	82.3% (370)	80.2% (329)	0.57
Assisting the patient by identifying behavior change strategies that will help achieve their goals	83.1% (368)	80.5% (330)	0.61
Recommending or referring the patient to weight management resources in the clinic or in the community	62.6 (263)	55.9% (209)	0.34
Recognizing opportunities during the clinical encounter to enhance patient confidence	74.8% (324)	72.0% (286)	0.50
Proposing that weight and weight management be discussed again at their next appointment	89.7% (384)	89.3% (350)	0.91
Demonstrating to the patient that you understand their perspective on weight management	78.7% (350)	76.8% (312)	0.64

### Intervention Tracking and Exposure Measures

Study coordinators tracked each student’s participation in the web course, role-play, and web-patient encounter. The total MME intervention exposure received per student was computed as the total number of components completed (0–3). MS3s at MME schools also completed a survey on participation in the Web course and role-play regarding WMC, The self-reported total intervention exposure was computed as the sum of students receiving each of the MME components (range 0–3).

### Statistical Analysis

Total OSCE case scores were analyzed using a two-stage mixed model analysis of variance (ANCOVA)^26^ . In stage 1, 16 (8 schools × 2 timepoints) means were estimated for student-level OSCEs. In stage 2, the eight post-intervention school means were regressed on randomization assignment and the corresponding pre-intervention school mean, adjusting the MME versus TE comparison for baseline school means and accounting for within-school correlation. In parallel, we estimated binomial logistic regression models for each of the OSCE items (completed versus not completed) as a function of randomization assignment with school as a random effect.^27^ Students in the MME intervention and students in the TE intervention (referred to below as MME students and TE students, respectively) were compared regarding perceived WMC skills using linear mixed modeling^28, 29^ for total score and binomial logistic regression for each of the 16 individual skills, with randomization assignment as a predictor and a random effect for school, adjusting for student’s MS1 score. In addition to our primary intention to treat analyses, we estimated associations of WMC training (“exposure”) with outcomes. For MME students, separate models were estimated with participation in each of the intervention components and number of intervention components as predictors. Combining MME and TE students, we estimated associations of outcomes with self-reported hours of WMC learning in MS1, MS2, and MS3. MME and TE students were also compared regarding self-reported hours using ordinal logistic regression with school as a random effect. To assess any impact of missing OSCE scores (29.9%, 390/1305), we multiply imputed missing OSCEs^30, 31^ using IVEware as a function of: randomization assignment, school, baseline school mean OSCE, gender, race, and MS1 and MS3 measures of attitudes towards WMC, obesity bias^32^, confidence in providing WMC, and perceived WMC skills. Results regarding MME-TE differences were consistent with those presented here (data not shown). Analyses used SAS 9.4^33^ and Stata 14.2^34^

## RESULTS

### Participating Students

**Figure 1.**
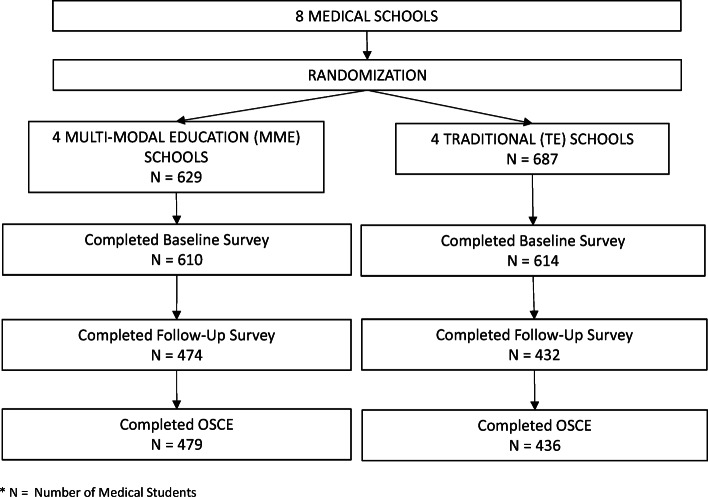
CONSORT/FLOW DIAGRAM for OSCE and Self-Reported Skills Outcomes

At MME schools, 629 MS3s were eligible; of these, 479 (76.2%) completed a third-year post-intervention OSCE, and 474 (76.3%) completed the follow-up survey. At TE schools, 687 MS3s were eligible; of these, 436 (63.5%) completed a third-year OSCE, and 432 (62.9%) completed the follow-up survey. Figure 1 shows reasons for missing outcomes. Among students providing OSCE data or self-reported WMC skills, MME and TE students were similar in age, race, Hispanic ethnicity, and gender. Students were ~24 years old, 51% were female, 74% identified as White, 6% as African American, 15% as Asian, 5% as Multi-racial, 1% as ‘other’ race, and 5% as Hispanic. Students reported their intended career as follows: primary care (24.1%), medical specialty (53.7%), other (11.4%), and undecided (10.8%)

### OSCE Outcomes

On average, MS3 MME students completed 8.7 of the 15 5A items on the case checklist (SE 0.11) versus 8.2 (SE 0.11) for TE students (p=0.16) (Table 1). MME students outperformed TE students on 12 of 15 items (p-values ranging from .09 to .91) . In both MME and TE schools, >85% of students reviewed medical risk factors and co-existing conditions of obesity, asked the patient standard questions about diet and physical activity, discussed the patient’s perceived barriers and concerns with losing weight/maintaining weight loss, and discussed behavior modifications to achieve their goals.

### Self-Reported WMC Skills

At post-intervention, mean total score (SD) was slightly higher for MME students (3.18) than TE students (3.09) (p=0.25, scale of 1 to 4). Although only one item-specific difference was statistically significant, a higher percentage of MME versus TE students reported feeling moderately/very skilled in 14 of the 16 WMC skills assessed (Table 2), with largest absolute differences for discussing the health benefits of losing weight, recommending or referring the patient to weight management resources, and advising weight loss based on their personal health information.

### WMC training in MME and TE students

At MME schools, more than 2/3 of students completed all three intervention-delivered activities (Table 3). MME students reported significantly more preclinical time in WMC during the MS1 and MS2 years than TE students, however both groups spent an equivalent amount of time during MS3.

**Table 3.** Exposure to WMC Training by Randomization Assignment

	**MME**	**TE**	
	**% (N)**	**% (N)**	**p-value**
**MME-delivered intervention components:**			--
Web course	74.3 (462)	--	
Role play	82.2 (511)	--	
Web patient encounter	76.4 (475)	--	
Number of intervention components:			
0	14.5 (90)	--	
1	6.1 (38)	--	
2	11.6 (72)	--	
3	67.9 (422)	--	
**Self-reported exposures:**			
During preclinical or 1^st^ and 2^nd^ years of medical school: Estimate of how much time was devoted to learning about weight management counseling.			.03
Less than 30 minutes	1.5 (7)	5.6 (24)	
30 – 59 minutes	4.2 (20)	7.0 (30)	
1 hour	4.4 (21)	8.6 (37)	
2 hours	14.6 (69)	25.1 (108)	
3 hours	15.9 (75)	20.7 (89)	
4 hours	18.2 (86)	14.9 (64)	
5 hours	10.6 (50)	5.6 (24)	
More than 5 hours	30.7 (145)	12.8 (55)	
During 3^rd^ year of medical school: Estimate of how much time was devoted to learning about weight management counseling.			.51
Less than 30 minutes	14.5 (65)	18.1 (78)	
30 – 59 minutes	10.7 (48)	10.2 (44)	
1 hour	17.4 (78)	16.7 (72)	
2 hours	20.6 (92)	15.6 (67)	
3 hours	11.0 (49)	14.1 (61)	
4 hours	8.3 (37)	6.7 (29)	
5 hours	4.3 (19)	3.7 (16)	
More than 5 hours	13.2 (59)	14.7 (63)	

### Associations of WMC training with outcomes

At MME schools, student participation in the three intervention components was positively associated with total OSCE score, with the largest difference for the web course (0.43 items difference on average), although the associations were not statistically significant (Table 4). MME students who completed two of the three components had the highest mean OSCE score at 9.02 (standard error 0.39), and those completing no components had the lowest mean OSCE score at 8.22 (standard error 0.44), although differences were not statistically significant (p=0.28). MME student participation in the three intervention components also was positively associated with perceived WMC skills, with statistically significant associations for all but web course participation. Among students at both MME and TE schools, time spent in WMC learning activities in the first two years ranging from 1 (<30 minutes) to 8 (>5 hours) was significantly positively associated with total OSCE score and total perceived WMC skills; an increase of 1 category (e.g., <30 minutes to 30-59 minutes) was associated with an average increase of 0.08 in the OSCE score and of 0.04 in total perceived WMC skills. The corresponding associations with time in MS3 year also were positive but smaller and not statistically significant for the OSCE score.

**Table 4.** Associations of WMC training with MS3 Total OSCE Scores and Perceived WMC skills

	Mean (SE)			
WMC training	Total OSCE score (range 0 – 15)	P-Value	Perceived WMC skills (range 1 – 4) ^(a)^	P-Value
*MME-delivered intervention components, MME students only:*				
Web course		0.11		0.21
Yes	8.82 (0.27)		2.96 (0.04)	
No	8.39(0.33)		2.88 (0.06)	
Role play		0.33		0.001
Yes	8.76 (0.28)		2.97 (0.02)	
No	8.44 (0.40)		2.77 (0.06)	
Web patient encounter		0.72		<0.0001
Yes	8.74 (0.30)		2.99 (0.03)	
No	8.61 (0.40)		2.76 (0.05)	
Number of components		0.28		0.002
0	8.20 (0.44)		2.77 (0.06)	
1	8.47 (0.50)		2.72 (0.09)	
2	9.02 (0.39)		2.99 (0.07)	
3	8.77 (0.29)		2.98 (0.03)	
	Slope coefficient (95% CI)			
*Self-reported hours devoted to WMC learning, MME and TE students:*				
Total hours in preclinical years 1 and 2 (range 1 – 8)	0.08 (0.01, 0.15)		0.04 (0.02, 0.06)	
Total hours in year 3 (range 1 – 8)	0.03 (-0.03, 0.09)		0.03 (0.01, 0.04)	

^(a)^Adjusted for student’s Year 1 perceived WMC score

## DISCUSSION

MME and TE students showed similar WMC skills assessed by OSCE and self-report. There were some suggestions of differences as MME students outperformed TE students on 12/15 OSCE items; however, this did not translate to a statistically significant higher overall score. Importantly, when data for MS1 and MS2 students from all schools were combined, an increased number of hours of training was significantly associated with both total OSCE score and total perceived WMC skills.

A likely explanation for the few differences between MME and TE students is that WMC training was implemented concurrently in TE schools, albeit in different forms than in the MME schools. While TE students reported fewer hours of WMC training compared to MME students in years 1–2, by year 3, the number of hours had equalized. The growing interest in obesity prevention and management likely influenced medical school training. TE schools were not restricted from enhancing their WMC curriculum and may have made changes unrelated to the MME intervention. Therefore, future studies should ensure that real-time assessments of the specific WMC curriculum occur in comparison/TE schools. There is the possibility that students perform better on WMC skills in the setting of the OSCE than they do in regular encounters with their patients. This raises the question of how real-time evaluation of student practice could be conducted.

An encouraging sign of the impact of the MME intervention is the higher OSCE scores and WMC self-reported skills among 67% of MME students who participated in most of the intervention activities versus MME students who did not, indicating that MME vs. TE differences could have been larger if more students had received the complete MME intervention. While three schools integrated MSWeight into their curriculum, one school made participation voluntary, suggesting that encouraging integration may have enhanced impact.

Notably, we found discrepancies between students’ perception of their skills and actual performance on the OSCE. Despite the majority of students in both groups self-reporting as moderately, or very skilled, in identifying and informing patients with obesity that their weight is a health concern, only a minority performed this task on the OSCE. A similar discordance was found for advising weight loss, which contrasts with our prior study in smoking cessation education where medical students showed high performance.^35^ Fewer students discussed weight history and prior weight loss experience during the OSCE despite having high self-reported skill in this area. Many clinicians feel uneasy broaching the topic of weight with their patients and may require more intensive training. While advising a patient to stop smoking is often seen as objective medical advice, advising patients to lose weight is more personal, and may even feel judgmental to the patient and provider. Similarly, asking patients about their weight history is more complicated than asking about smoking history. Therefore, while students (and providers) know they should discuss weight issues with patients, implementation is more complicated.

Our findings suggest that students need more information regarding training on the specific details of nutrition and physical activity. Few students provided specific information on the health benefits of weight loss or the relationship between weight, diet, and physical activity, suggesting that while they performed well in general behavioral change counseling, they struggled to provide specific guidance. It is possible that students were unaware of this information, highlighting a curriculum gap in education about nutrition and exercise as well as a gap in how to deliver such information.

### Strengths and Limitations

The major strengths of this study were the randomized design, the participation and excellent response rates of eight schools nationwide, high-fidelity across multiple and diverse medical schools, relatively strong student participation in all MME components, and rigorous evaluation via two time-tested assessments (OSCE and self-report). We also chose schools that had ≤4 hours of WMC education to attract schools without a WMC-rich curriculum.

Because only eight U.S. medical schools were involved, the study may have limited generalizability. A second concern is that questions do not capture the full breadth of student performance,for example, students may conduct routine WMC but are unaware of doing this for patients with co-existing conditions like diabetes and heart disease. Third, while there is some concern that one-third of students did not complete follow-up assessments, most missing data in both arms was attributed to academic reasons (e.g., dismissed / left school, schedule shift, delayed by a semester,change of campus, part of MD/PhD program, leave of absence). Finally, while OSCE’s are widely used in medical education, performance on a single OSCE in this case as part of the existing curriculum provides only a snapshot of a student’s skill in WMC.

### Conclusion

This study is consistent with our prior trial to improve medical students’ tobacco treatment counseling skills.^35^ In both cases, students in TE schools received enough training to possibly dilute an effect from the MME curriculum. Unlike our prior study, where students performed well at advising patients to stop smoking, this study showed that students struggle with advising patients about diet, exercise, and the benefits of weight loss and weight management.

A systematic review in 2020 of weight-related communication trainings for physicians concluded that physician trainings should be grounded in a theoretical framework and emphasize patient-centered communication—experiential learning and skill development should be central components since they appear to improve physician outcomes^36^. Our results are in line with these recommendations. Future WMC curricula must improve instruction in nutrition and exercise, along with behavioral change counseling skills. Experiential education to provide knowledge and build confidence in delivering counseling should start early in medical school and be boosted throughout the four years of training.

We acknowledge all school site PIs, research coordinators, evaluators, directors of standardized patients, medical directors of clinical education and simulation center, research assistants, support staff, participating medical students, course directors, clerkship directors, preceptors and our weight bias consultant who have and will implement and participate in the study.
